# Genetic diversity and signature of divergence in the genome of grapevine clones of Southern Italy varieties

**DOI:** 10.3389/fpls.2023.1201287

**Published:** 2023-09-13

**Authors:** Clizia Villano, Silvia Procino, Giuseppe Blaiotta, Domenico Carputo, Nunzio D’Agostino, Ermanno Di Serio, Valentina Fanelli, Pierfederico La Notte, Monica Marilena Miazzi, Cinzia Montemurro, Francesca Taranto, Riccardo Aversano

**Affiliations:** ^1^ Department of Agricultural Sciences, University of Naples Federico II, Portici, Italy; ^2^ Department of Soil, Plant and Food Sciences, University of Bari Aldo Moro, Bari, Italy; ^3^ Institute of Biosciences and Bioresources (CNR-IBBR), Bari, Italy; ^4^ Support Unit Bari, Institute for Sustainable Plant Protection, National Research Council of Italy (CNR), Bari, Italy; ^5^ SINAGRI S.r.l., Spin Off of the University of Bari Aldo Moro, Bari, Italy

**Keywords:** *Vitis vinifera*, intra-varietal diversity, genotyping by sequencing, double digest restriction associated DNA, molecular markers, divergent loci

## Abstract

Sexual reproduction has contributed to a significant degree of variability in cultivated grapevine populations. However, the additional influence of spontaneous somatic mutations has played a pivotal role in shaping the diverse landscape of grapevine agrobiodiversity. These naturally occurring selections, termed 'clones,' represent a vast reservoir of potentially valuable traits and alleles that hold promise for enhancing grape quality and bolstering plant resilience against environmental and biotic challenges. Despite their potential, many of these clones remain largely untapped.In light of this context, this study aims to delve into the population structure, genetic diversity, and distinctive genetic loci within a collection of 138 clones derived from six Campanian and Apulian grapevine varieties, known for their desirable attributes in viticulture and winemaking. Employing two reduced representation sequencing methods, we extracted Single-Nucleotide Polymorphism (SNP) markers. Population structure analysis and fixation index (FST) calculations were conducted both between populations and at individual loci. Notably, varieties originating from the same geographical region exhibited pronounced genetic similarity.The resulting SNP dataset facilitated the identification of approximately two hundred loci featuring divergent markers (FST ≥ 0.80) within annotated exons. Several of these loci exhibited associations with essential traits like phenotypic adaptability and environmental responsiveness, offering compelling opportunities for grapevine breeding initiatives. By shedding light on the genetic variability inherent in these treasured traditional grapevines, our study contributes to the broader understanding of their potential. Importantly, it underscores the urgency of preserving and characterizing these valuable genetic resources to safeguard their intra-varietal diversity and foster future advancements in grapevine cultivation.

## Introduction

According to the data from the Food and Agriculture Organization (FAOSTAT, 2023), Italy holds a significant position among the leading grape-producing nations. This prominence can be attributed to several unique factors, all encapsulated in the concept of “terroir”. The Italian wine industry is variegated due to the distinct characteristics of the regions, including their physical, climatic, and cultural aspects. However, the biggest contributing factor is the availability and use of a wide range of grapevine varieties cultivated in different part of the Country (Sardaro et al., 2017; Pomarici et al., 2021).

To date, based on the Italian Vitis database (talian Vitis Database, 2023; D’Onofrio and Scalabrelli, 2010) and the National Register of Grapevine Varieties (http://catalogoviti.politicheagricole.it) more than 1,100 varieties have been collected and described; however, the actual number of grape varieties cultivated and kept in germplasm collections in Italy is much higher and cannot be estimated with certainty. The invaluable germplasm of Italian grapes includes many autochthonous varieties. Although sexual reproduction has led to a great variability among varieties (*i.e.*, inter-varietal diversity), spontaneous somatic mutations have further contributed to the kaleidoscope of grapevine diversity with the so-called clones (Salmaso et al., 2005). As a result, autochthonous varieties are made up of groups of different clones with genotypic, morphological, and physiological characteristics slightly different from those of the original mother plant (*i.e.*, intra-varietal diversity). An effective approach to explore the inter- and intra-varietal diversity relies on the use of molecular markers. Microsatellites are the most used markers in grapevine, and a standard set of nine loci is currently used for international cataloguing (This et al., 2004; Laucou et al., 2011; Vitis International Variety Catalogue VIVC, 2023). Single nucleotide polymorphisms (SNPs) are another class of markers widely used for grape genotyping, which promise a higher map resolution, higher throughput, lower cost, and lower error rate than microsatellites (Vezzulli et al., 2008; Kumar et al., 2012; Vishwakarma et al., 2022). Analyses of SNPs variations can only be conducted in a small fraction of the genome with Reduced Representation Sequencing (RRS) (Pavan et al., 2020). The most popular techniques use restriction enzymes to prepare DNA for sequencing (Restriction-site-Associated DNA: RAD). Many methods based on the RAD approach have been developed in recent years, differing in the number of enzymes used or in additional library preparation steps (Andrews et al., 2016). In plant, the most used are GBS (genotyping-by-sequencing), which generally relies on a single restriction enzyme (Elshire et al., 2011) and ddRADseq (double-digest RAD sequencing), in which DNA is digested with two restriction enzymes (Peterson et al., 2012; O'Leary et al., 2018). Both GBS and ddRADseq techniques have been successfully used in *Vitis* genotyping to study intra-varietal diversity (Miazzi et al., 2020; Calderón et al., 2021; D’Onofrio et al., 2021).

Southern Italy is recognised as the oldest wine-growing area in Italy where many traditional and autochthonous varieties are still cultivated (De Lorenzis et al., 2019). They represent a vast reservoir of traits/alleles that could be useful for improving the quality of grape as well as plant tolerance to environmental and biotic stresses. However, most of them are still underexploited. In this framework, the present study is aimed to analyse population structure, genetic diversity, and divergent loci in a panel of 138 clones belonging to six Campanian and Apulian grapevine varieties characterised by attractive traits from a viticultural and oenological point of view. To genotype this plant material, we used ddRADseq and GBS techniques on Campanian and Apulian clones, respectively. The analysis revealed the complex genetic structure of the varieties under investigation at the clonal level, their relationships, and the presence of divergent SNP loci within genes involved in grape phenology and adaptation to the environment.

## Materials and methods

### Plant material

**Figure 1:**
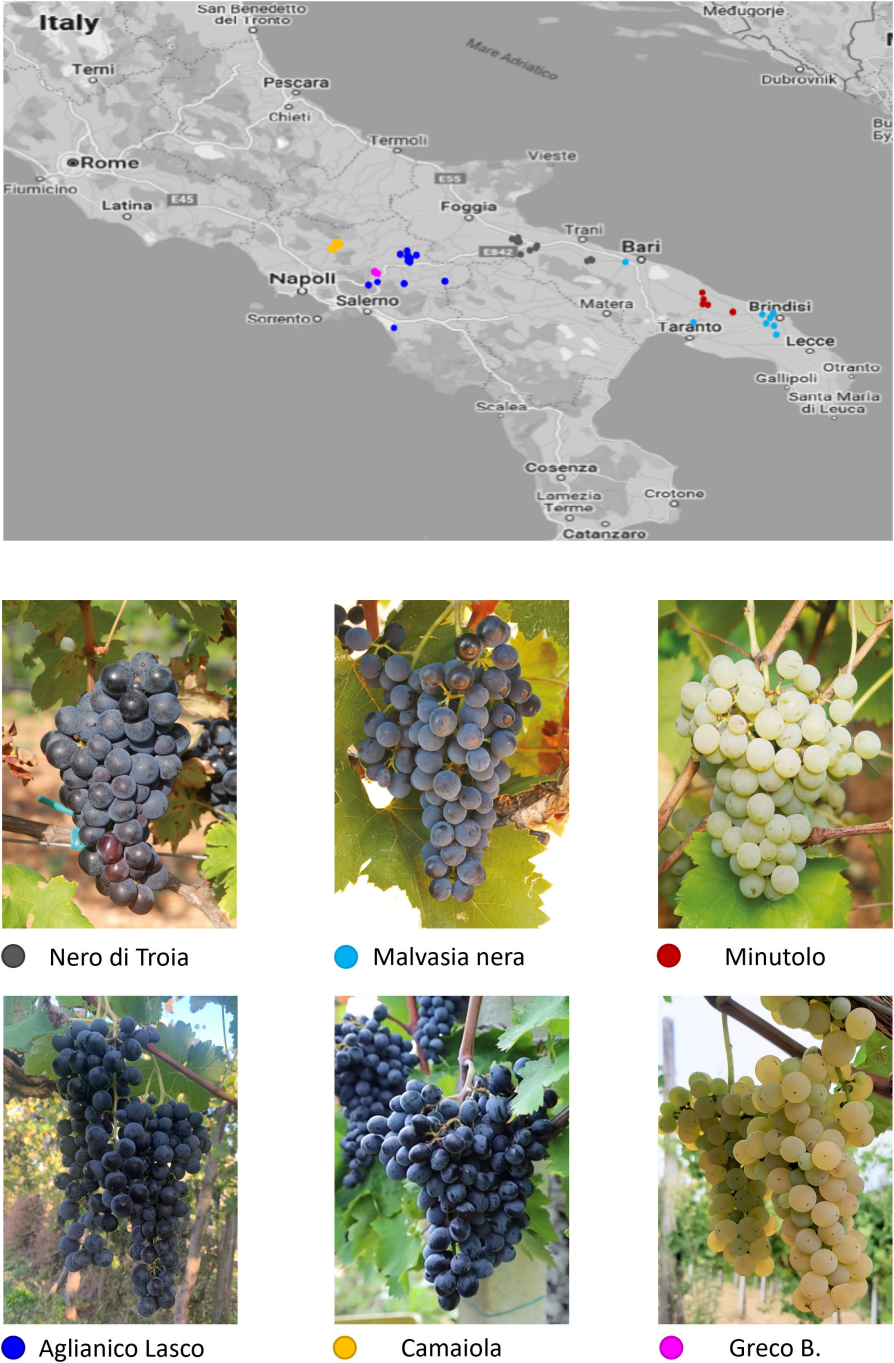
Map of southern Italy with the sampling sites for the Campanian and Apulian clones of the six varieties under investigation.

A total of 138 grapevine clones belonging to 6 autochthonous varieties of Southern Italy were sampled in Campania and in Apulia (**Supplementary Table S1**). They are traditionally grown in environments with different climate conditions: plains with hot, dry summers and mild winters for the Apulian varieties; hills with warm, humid summers and cold winters for the Campanian varieties. The Campanian clones include two red-berry varieties, camaiola (formerly barbera del sannio) (n. 20) and aglianico lasco (n.64), and the white-berry greco b. (n. 24). aglianico lasco has loose clusters able to reduce the development of mold during ripening. camaiola has intense ruby red colour berries suitable for violet hues wines, and greco b. fruits give rise to high quality wines appreciated for the complex aroma profiles (Antonacci et al., 2017). The Apulian clones belong to two red-berry varieties, nero di troia (synonym uva di troia; n. 16) and malvasia nera di brindisi (hereafter referred as malvasia nera; n. 8), and to the white-berry minutolo b. (n. 6). nero di troia is a high-quality, flavourful variety cultivated mainly in northern Apulia to make an ever-increasing number of monovarietal wines or blends. malvasia nera is a variety of Greek origin, widely cultivated in the southern part of Apulia, which produces fruity wines often together with the local variety negramaro. minutolo b. is an aromatic white variety with relatively small and loose clusters, which in the past was widely grown in the specific area of Apulia region called Itria Valley. The map of the sampling sites is shown in **Figure 1**. No major morphological and physiological differences were found among the clones of the six varieties analysed. In addition, four native (aglianico del vulture, aglianico taurasi, aglianico del taburno and sangiovese) and two international (chardonnay and merlot) varieties were used for microsatellite (SSR) analysis.

### DNA isolation

As for Campanian clones, the total DNA was extracted from 1 gr of young leaf tissue following the method by Japelaghi et al. (2011) with a few modifications, such as a ratio of 0.2 g tissue/1 mL extraction buffer, an increase in the concentration of soluble PVP from 2% (w/v) to 4% (w/v) in the extraction buffer, and an additional washing step with 70% (v/v) ethanol (EtOH) before elution.

As for Apulian samples, the DNA was extracted following the procedure described in Spadoni et al. (2019). For all samples, the yield, quantity, and quality of extracted DNA were estimated using a NanoDrop 1000 Spectrophotometer (Thermo Scientific, Wilmington, DE, USA), a 0.8% agarose gel, and a fluorimeter (Qubit 2.0, Thermo Fisher Scientific) with the Qubit dsDNA BR Assay kit (Invitrogen, CA, USA).

### SNP calling

Campanian genetic material was genotyped using ddRADseq (Peterson et al., 2012). The ddRADseq tags were aligned to the reference genome GCA_000003745.2 (Pinot Noir, PN40024) using BWA-MEM (Li and Durbin, 2009). The *gstacks* and *populations* (with options -R = 0.75 and –max-obs-het = 0.8) utilities included in Stacks v2.53 (Catchen et al., 2013) were used for SNP calling and for applying filtering options. Genotyping-by-sequencing and SNP calling on Apulian samples were performed by Elshire group Ltd. (https://www.elshiregroup.co.nz/) as described in Miazzi et al. (2020), using the same reference genome as above.

### SNP filtering and statistics

ddRADseq and GBS produced two Variant Call Format (VCF) files respectively for Campanian and Apulian varieties, which underwent SNP filtering using VCFtools v. 0.1.16 (Danecek et al., 2011). For these two datasets, SNP markers with minor allele frequency (MAF) < 5%, minimum site count < 15%, and a minimum depth of coverage of 5 were filtered out. Instead, for the dataset of each variety, SNP markers with a call rate of 100%, minor allele frequency (MAF) < 5%, and a minimum depth of coverage of 5 were filtered out. The choice of not having missing data was necessary for the intra-varietal analysis, as the missing data affected the Identity-by-State (IBS) values. VCFtools were also used to generate various statistics on the datasets under investigation and to add gene annotations to VCF files.

### Identity-by-State and linkage disequilibrium analysis

PLINK v.1.90 (Purcell et al., 2007) was used to obtain the IBS distance matrix for each variety. Duplicate individuals were identified by setting IBS value ≥ 0.99 as the threshold. Only one individual was retained among duplicates and used for downstream analyses.

For each dataset, linkage disequilibrium (LD) analysis was performed using the Golden Helix SNP and Variation Suite (SVS) v.8.8.3 (Golden Helix Inc.). The “Nonlinear Regression” function was used to plot the r^2^ values against the physical distance of the markers, and the LD decay was estimated at the critical level of r^2 = ^0.20.

### Merging SNP data points

After removing the duplicates, bcftools +fixref (Li, 2011) was used to fix the inconsistencies in reference (REF) – alternative (ALT) alleles between the two VCF files. Then the *vcf-merge* utility of VCFtools (Danecek et al., 2011) was used. VCFtools (Danecek et al., 2011) and PLINK (Purcell et al., 2007) were used to filter out individuals with SNP markers with a missingness per individual > 85%, MAF < 5% and missingness per marker >20%. The kinship coefficient was calculated using the *–relatedness2* option in VCFtools. The dataset was LD pruned (r^2 = ^0.50) using SVS.

### Genetic diversity and population structure

Allele frequency and ancestry estimation were performed on filtered and pruned dataset using ADMIXTURE v. 1.3.0 (Alexander and Lange, 2011), with 10-fold cross validation (CV) for sub-populations (K) ranging from 1 to 10, and 1,000 bootstrap replicates. CV scores were used to estimate the optimal K value. A membership coefficient (qi) >0.55 was used to separate the individuals into sub-populations. Discriminant Analysis of Principal Components (DAPC) was used as an exploratory data analysis to investigate population structure (Jombart et al., 2010; Jombart and Ahmed, 2011). The optimal number of principal components (PCs) to maintain was determined using a value ≥ 1:200. The optimal number of k-means was determined using the Bayesian information criterion (BIC) as a statistical measure of goodness-of-fit. Principal component analysis (PCA) was performed using SVS and Neighbor-joining trees (bootstrap = 1,000) were built with MEGA v.11. FigTree v.1.4 was used to visualise trees (Rambaut, 2014; http://tree.bio.ed.ac.uk/software/figtree; Tamura et al., 2021). Haplotype diversity and analysis of molecular variance (AMOVA) was conducted using GenALEx version 6.5 (Peakall and Smouse, 2006) to calculate variance components and their levels of statistical significance for variation between and within populations.

### Microsatellite analysis

Total genomic DNA was extracted from young leaves of Aglianico Lasco clones using the Qiagen Plant DNeasy Maxi Kit (Qiagen, Valencia, CA, USA), following the manufacturer’s procedure. Microsatellite analysis was carried out with seven nuclear markers (VVMD27, VVlb01, Vvln16, VVIp60, VVIq52, VrZAG79, and VVS2) from Laucou et al. (2011). PCR amplification, size calibration, alleles detection was performed as reported by Villano et al. (2014). Validation of results was performed with three biological and technical replicates. Allele sizes were normalised using SSR data reported in the Vitis International Variety Catalog (http://www.vivc.de/). The phylogenetic clustering tree was constructed with the neighbour joining method using MEGA X (Tamura et al., 2021). The robustness of the clusters was tested by bootstrap resampling (n = 1,000) with the Darwin software (Perrier et al., 2003).

### Signature of divergence

Per-site F*_ST_* calculation was performed on the filtered dataset using SVS and comparing pairs of populations each time as described in Taranto et al. (2020). Only divergent SNP markers (F*_ST_* ≥ 0.80) within annotated exons were considered for the search for candidate genes. Grapevine gene annotations were retrieved at https://urgi.versailles.inra.fr/Species/Vitis/Annotations.

## Results

### Intra-varietal diversity: genome-wide SNPs discovery and the extent of linkage disequilibrium

A total of 114,465 and 180,840 SNP markers were scored in the 108 Campanian and in the 30 Apulian clones, respectively. After filtering, 59,149 and 17,296 SNPs were retained in the Campanian and Apulian datasets, respectively (**Table 1**; **Supplementary Figures 1A, B**). Transitions (Ti) were more abundant (81%) in the Campanian dataset than in Apulian (63.33%), with a Ti/Tv ratio of 4.22 and 1.72, respectively. Taking advantage of the genomic coordinates of the reference gene models, 15,611 (26.4%) and 8,877 (51.3%) SNPs fell within annotated exons for Campanian and Apulian datasets, respectively. The number of SNPs before and after filtering and the statistics of high-quality SNPs are shown in **Table 1**.

**Table 1:** Number of SNPs before and after filtering procedure and statistics of high-quality SNPs calculated for each dataset.

Dataset	#SNPs before filtering	#SNPs after filtering	#SNPs within genes	Transitions (Ts)	Transversions (Tv)	Ts/Tv ratio
Apulia	180,841	17,296	8,877	10,953	6,343	1.72
Campania	114,465	59,149	15,611	47,832	11,317	4.22
Aglianico Lasco	114,465	9,061	2,966	7,426	1,635	4.54
Camaiola	114,465	10,930	3,248	8,983	1,947	4.61
Greco Bianco	114,465	9,899	3,044	8,125	1,774	4.58
*Average*		9,963	3,086	8,178	1,785	5
Malvasia	180,841	15,883	7,643	9,964	5,647	1.76
Minutolo	180,841	7,408	5,511	4,708	2,648	1.77
Nero di Troia	180,841	5,094	3,746	3,271	1,781	1.83
*Average*		9,462	5,633	5,981	3,359	2
Average (Total)	147,653	9,712.50	4,359.60	7,079.50	2,572	3.18

Transitions are interchanges of A/G and C/T bases, whereas transversions are interchanges of A/C, A/T, G/T and C/G bases.

The clones of each variety were separated into six VCF files and each dataset was subjected to the filtering procedure. After filtering, 9,963 and 9,461 (on average, avg) SNPs were retained in the Campanian and Apulian datasets, respectively.

Of these SNPs, an average of 3,086 fell within annotated genes in the Campanian datasets and 4,544 in the Apulian ones. Malvasia showed the greatest number of SNPs within genes (N = 7,644), while Greco B. showed the least (N = 2,966). In all the varieties there were more transitions (Ts) (7,079 avg) than transversions (Tv) (2,572 avg) and the average Ts/Tv ratio was higher in the Campanian varieties (4.60) than in the Apulian ones (1.80) (**Table 1**).

The high-quality SNP datasets were then used to calculate the pairwise IBS distance between clones of each variety. Clone pairs with an allele sharing rate > 99% were considered duplicates and only one of them was retained for downstream analysis. No duplicate clones were found in Camaiola and Apulian germplasm, whereas 47 and 13 duplicates were removed in Aglianico Lasco and Greco B., respectively, resulting in a final number of 78 clones ( **Supplementary Table S1**). The LD decay was estimated for each dataset. At r^2 = ^0.20, Aglianico Lasco showed the slower LD decay of 254.3 kb. In contrast, Camaiola, Greco B., Minutolo, Malvasia and Nero di Troia were characterised by very rapid LD decay (< 0.093kb) (**Supplementary Figure S2**).

### Inter-varietal diversity: population structure and F_ST_

**Figure 2:**
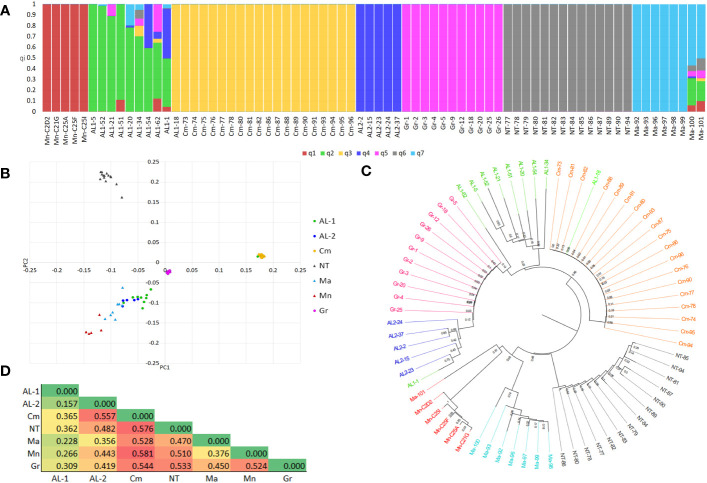
**(A)** Bar-plot describing the population structure estimated by ADMIXTURE. Each bar is divided into K coloured segments each representing the proportion of ancestry (q_i_) in each individual. **(B)** PCA plot of 72 grapevine clones obtained using 1,091 SNPs. **(C)** Neighbour-Joining tree. **(D)** Heatmap of fixation indices (*F_ST_*) between subpopulations.

The 78 clones retained after IBS analysis were merged into a single dataset of 2,235 SNP markers. After filtering, 1,091 high-quality SNPs and 72 individuals were retained (**Supplementary Table S1**). The distribution of SNPs along the 19 chromosomes is shown in **Supplementary Figure S3**. The dataset was further pruned for LD (r^2 = ^0.50) resulting in 500 SNPs, out of which 280 fall within genes. Population diversity was analysed using different approaches. The population structure indicated K = 6 and K = 7 as the best numbers of sub-populations, based on cross-validation error (**Supplementary Figure S4**). At K7 (**Figure 2A**), all the varieties grouped their own clones into separate sub-populations, with the exception of Aglianico Lasco which was separated into two sub-populations (henceforth AL1 and AL2), comprising nine and four clones, respectively. Individuals belonging to the sub-populations Minutolo, Camaiola, AL2, Greco B. and Nero di Troia shared a co-ancestry coefficient (q_i_) > of 0.99, whereas the clones of AL1 and Malvasia had greater variability with q_i_ ranging from 0.99 to 0.60 and from 0.99 to 0.57, respectively. The clones AL1-62, AL1-1, Malvasia-100 and Malvasia-101 resulted admixed. In addition, clone AL1-18 was grouped with clones belonging to Camaiola instead of Aglianico Lasco. DAPC analysis was then performed which confirmed the existence of seven clusters (**Supplementary Figure S5**) albeit with a slightly different clone distribution (**Figure 2B**). The Nero Di Troia and Camaiola clones were alone in the first and second quadrant, respectively. The Greco B. clones were grouped in the centre of the plot, while those of Aglianico Lasco (AL1 and AL2) were scattered in the fourth quadrant together with Malvasia Nera and Minutolo. Inter-varietal structuring within clones was supported by the neighbour-joining tree (**Figure 2C**). The phylogenetic analysis highlighted the geographical differentiation of the clones. The Apulian and Campanian clones formed well distinct clusters within which the clones belonging to each variety formed well-supported subclades. Also, the clustering of Aglianico Lasco confirmed the results of ADMIXTURE. These clones were divided into two subclusters, except for AL1-1, AL1-18, and AL1-62 which fell respectively in AL2, Camaiola and Greco B.

The analysis of haplotype diversity corroborated these results, in fact Camaiola and Greco B. showed lower values (h =0.041 and 0.058, respectively) than AL1 (h = 0.427), AL2 (h = 0.456), Minutolo (h = 0.173), Nero Di Troia (h = 0.236) and Malvasia Nera (h = 0.350). This was supported by the within-population sum of squares (SSWP) from the AMOVA analysis, where SSWP was 349.636 and 423.789 for Greco B. and Camaiola, respectively. The highest SSWP was found in AL1 (2097.001). Additionally, the AMOVA revealed high genetic variability within (41%) and between (59%) populations. Further investigation of genetic distances was performed using the genetic fixation index (F*_ST_*) between sub-populations as emerged from clustering of ADMIXTURE and DAPC (**Figure 2D**). The heatmap in **Figure 2D** shows that all varieties are genetically distinct. The greatest distance was found between Camaiola and all the other populations (F*_ST_* mean value > of 0.52), with the highest value detected between Camaiola and Minutolo/Nero di troia (F*_ST_ =* 0.58), followed by Nero di Troia with F*_ST_* mean value > 0.48. Clones belonging to AL1 showed the lowest mean values (*F*_ST_ = 0.27) with lower *F*_ST_ (0.16) detected with AL2.

### Genetic distance of AL1 and AL2 sub-populations based on IBS and microsatellite analyses

An additional intra-variety IBS and Kinship analysis was performed for AL1 and AL2 individuals. The pairwise genetic distance range was different within the two populations, 0.61-0.70 for AL1 and 0.78-0.99 for AL2. IBS values calculated between AL1 and AL2 individuals showed that IBS ranged from 0.53 to 0.73.

**Figure 3:**
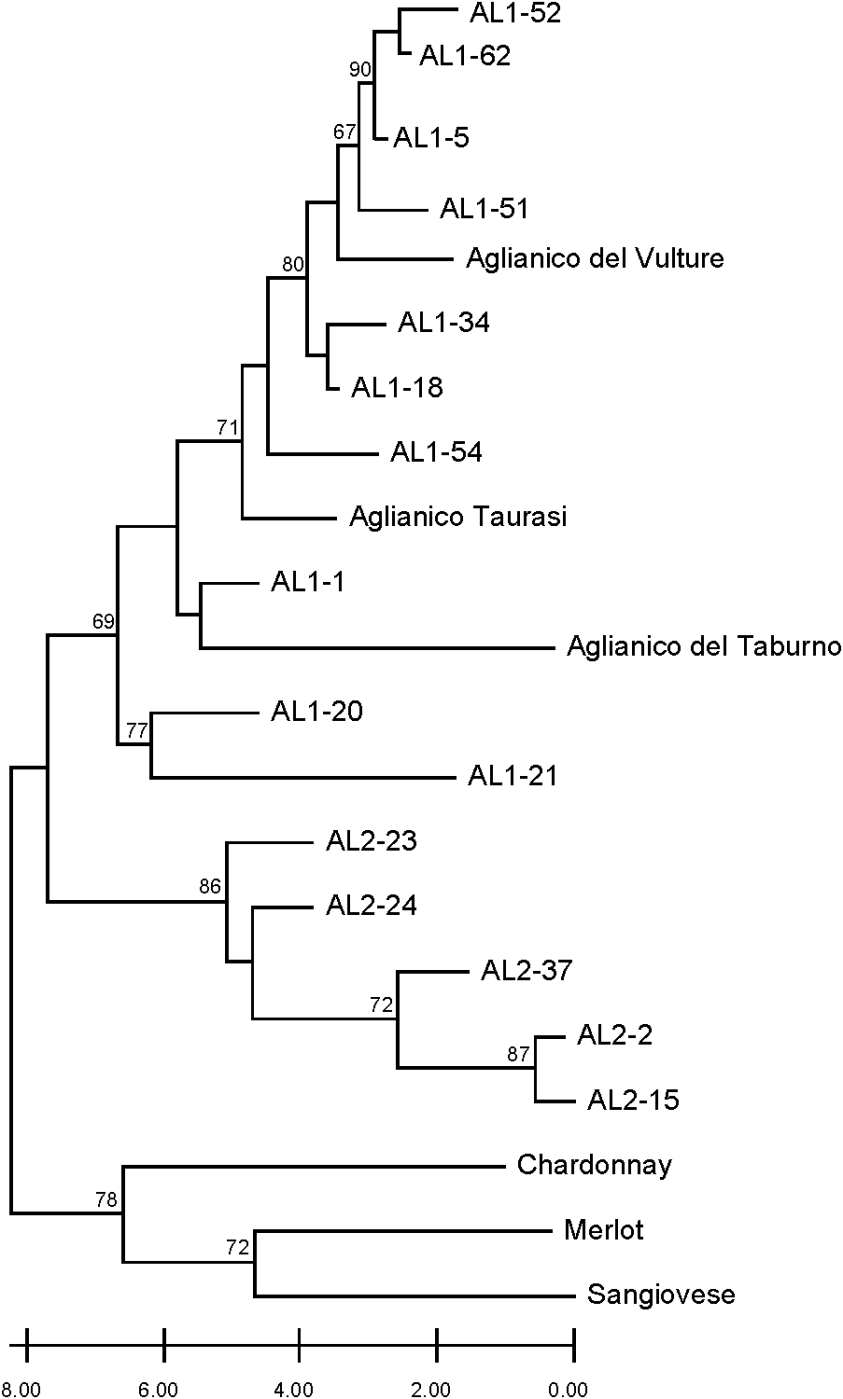
Dendrogram generated by neighbour-joining clustering of SSR markers for clones AL1, AL2, and Aglianico, as well as Sangiovese, Chardonnay, and Merlot. Bootstrap values greater than 60% are indicated.

The separation of Aglianico Lasco into two populations (namely AL1 and AL2) was further investigated using a set of seven discriminating microsatellite markers and the varieties Aglianico del Vulture, Aglianico Taurasi, Aglianico del Taburno, Sangiovese, Chardonnay and Merlot. The neighbour joining tree differentiated two main clusters (**Figure 3**). The first included all international varieties and Sangiovese, the other included AL1, AL2 and all Aglianico biotypes. In the latter, the AL1 clones were grouped together with Aglianico del Vulture, Taurasi and Aglianico del Taburno, while the AL2 clones were clearly grouped in a separate sub-cluster where no other varieties were included.

### Identification of divergent loci

**Figure 4:**
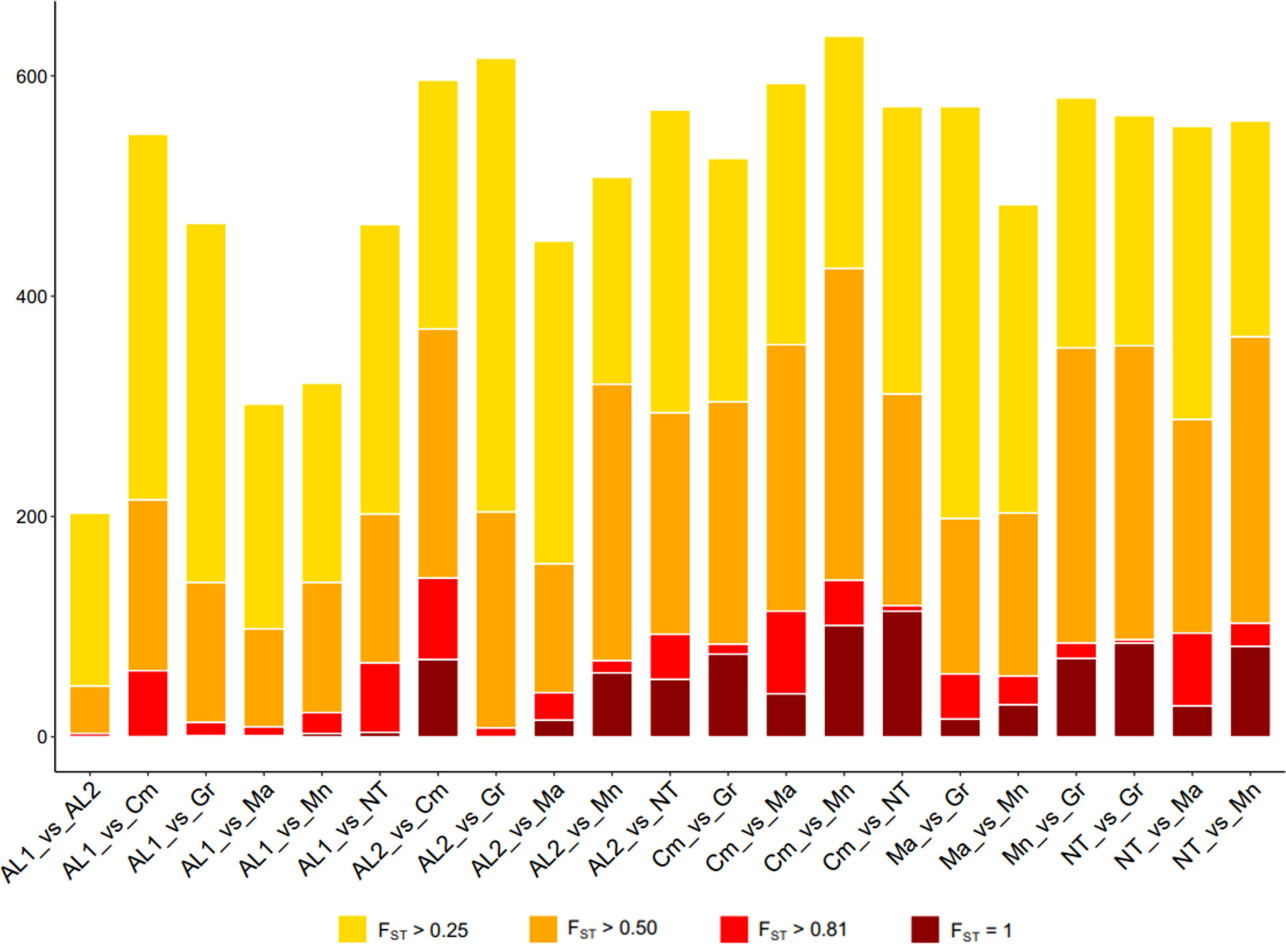
Number of SNPs with *F_ST_* > 0.25 (yellow), *F_ST_* > 0.5 (orange), *F_ST_* > 0.8 (red) and *F_ST_* =1 (darkred) detected in each comparison by computing pairwise *F_ST_* index between all loci.

**Figure 5:**
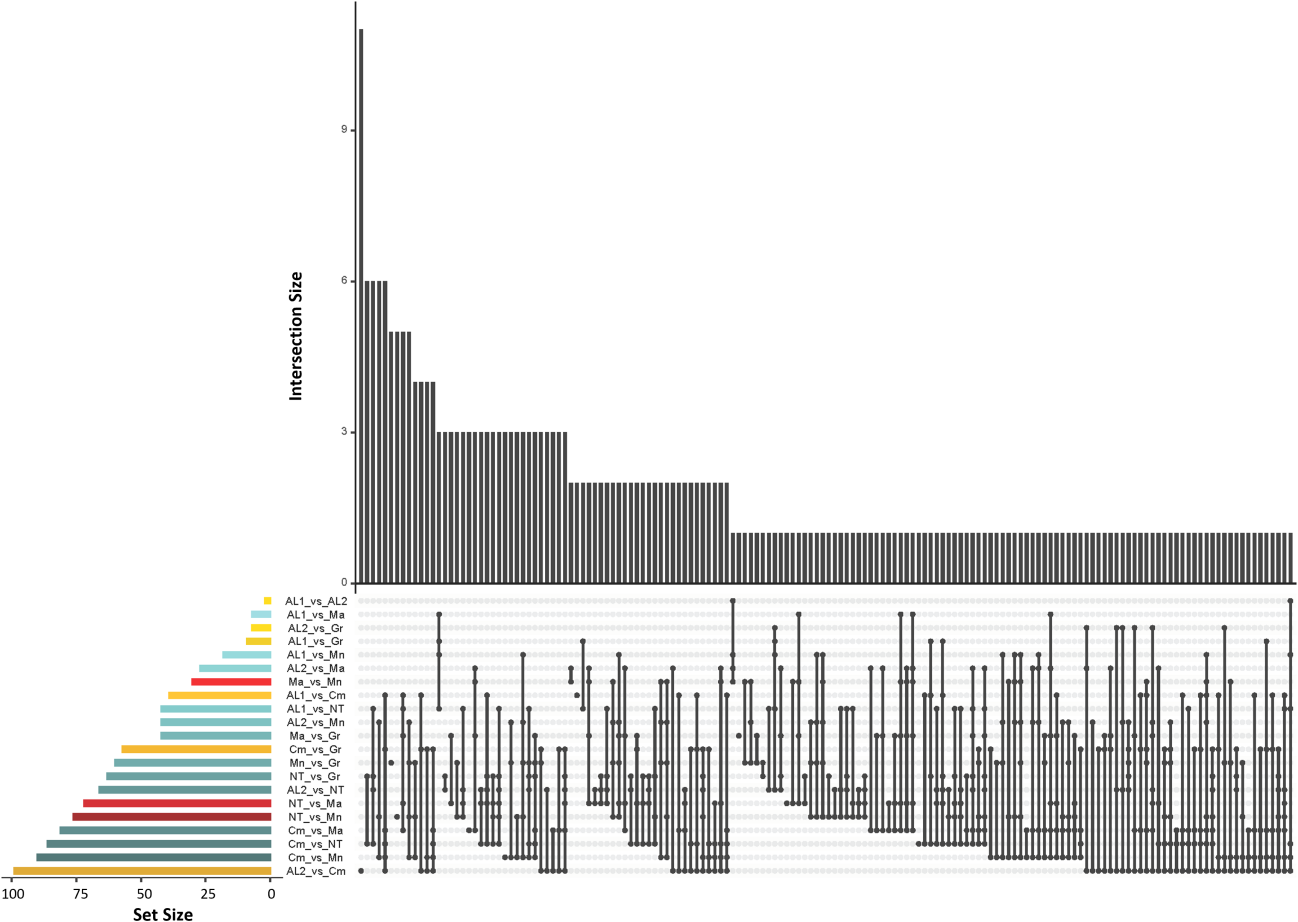
UpSet plot showing the number of the co-occurence of divergent SNPs (*F_ST_* ≥ 0.80) shared between the seven populations. The dot-and-line chart in the bottom combination matrix indicates intersections between the seven populations. The upper bar chart (black bars) indicated the numbers of divergent loci in each set; The lower left horizontal bar chart (coloured bars) indicated the number of divergent loci for each intersection. Different colours represent comparisons of Apulia vs Apulia varieties (red), Campania vs Campania varieties (yellow) and Apulia vs Campania varieties (blue).

Genetic differentiation among the seven populations described above was further investigated by Wright Fixation Index (F*_ST_*) analysis at individual loci. All comparisons revealed numerous loci with an F*_ST_* value > 0.25 (high differentiation) (**Figure 4**). To look for strongly divergent loci, F*_ST_* ≥ 0.80 was used as a threshold, resulting in 1,016 divergent SNPs, out of which 285 SNPs were non redundant. Among the latter, 200 were in gene regions, and their distribution along the chromosomes was reported in **Figure S6** and **Supplementary Table S3**. The largest number of divergent SNPs was found in Camaiola (N. = 453) followed by Nero di troia (N. = 404) and Minutolo (N. = 317), while AL1 showed the lowest number (N. = 117). An upset plot showing the co-occurrence of pairwise divergent loci is shown in **Figure 5**. As indicated by the “set size” in the **Figure 5**, Camaiola had the largest number of divergent genes between the other varieties, 100 against AL2, 91 against Minutolo, 85 against Nero di Troia, 81 against Malvasia, 57 against Greco B. and 39 against AL1 (**Supplementary Table S3**). In total, 81% of divergent genes were found in Camaiola comparisons. On the counterpart, AL1 and AL2 showed only two divergent genes (**Supplementary Table S3**), which represented the lowest number among all comparisons. To further our understanding of the 200 gene-associated divergent SNPs, we annotated the corresponding loci and categorised them into 14 major groups (e.g., development, hormone signalling, primary and secondary metabolism, stress response, etc.) (**Supplementary Table S4**). The most represented group was the “primary metabolism” with 62 genes, followed by “cellular component organisation and biogenesis” (28 genes), and “regulation of gene expression” (24 genes). The largest number of divergent SNPs (N=5) were found in VIT_214s0060g02480 (mainly identified in Malvasia comparisons), VIT_215s0021g01230 and VIT_212s0059g02300 (only in Nero di Troia vs MINUTOLO) and VIT_205s0029g01285 (mostly found in Nero di Troia comparisons), which are involved in “cellular component organisation and biogenesis”, “primary metabolism”, “regulation of gene expression” and “stress response”, respectively. Additional information associated with the 200 gene-associated divergent SNPs was retrieved from published data and allowed the identification of 63 SNPs associated with genes involved in grape phenology and adaptation to the environment (**Supplementary Table S4**). Among the latter genes, VIT_204s0008g01910 (mainly in Malvasia and Nero di Troia comparisons) and VIT_215s0048g02790 (mostly in Camaiola comparisons) were the most enriched in divergent SNPs. They are annotated as ferredoxin-related gene and PMT26 methyltransferase, respectively, and known to be linked to the initiation of berry ripening (**Supplementary Table S4**).

## Discussion

### The combination of ddRADSeq and GBS leads to a single high-quality SNP dataset

Six varieties were genotyped with two RRS strategies; GBS was used for the Apulian clones, while ddRADSeq for the Campanian ones. These methods differ in the number and type of restriction enzymes (REs) used to digest and access the genome and affected the total number of SNPs spanning on the reference genome. As expected, the ddRAD-seq dataset has more SNPs compared to fewer SNPs from GBS data, due to more restriction sites that are more polymorphic than GBS (Ruperao et al., 2023). The RE affected also the number of SNPs detected in genic and intergenic regions, indeed, the RE used for the Apulian dataset was *Ape*K1, a methylation-sensitive RE showing increased cleavage frequency in single-copy genomic regions that are enriched with genes (Elshire et al., 2011). Otherwise, a double digestion with a pair of rare and frequent cutter REs (*Sph*I and *Mob*I) performed for samples from Campania, according to the method proposed by Peterson et al. (2012) and modified by Scaglione et al. (2015), showed higher number of SNPs in intergenic regions. To address this challenging task, we searched for and found common SNP markers between the Campania and Apulia datasets and corrected for discrepancies using an *in silico* approach. Remarkably, after removing duplicate clones from each variety, we successfully merged all clones into a single dataset consisting of 2,235 SNP markers that was then used for cross-varietal analysis. To date, only Guillardín-Calvo et al. (2019) used both genotyping methods in Mediterranean evergreen oaks for technical comparison purposes. To the authors’ knowledge, this is the first time that ddRADSeq and GBS datasets have been merged and used to analyse the structure and genetic diversity of grapevine populations. Following this approach, we provide a consistent and repeatable framework that not only streamlines computational analyses, but also facilitates more general comparisons between different datasets, adhering to ‘FAIR’ (Findability, Accessibility, Interoperability, and Reusability) principles (Wilkinson et al., 2016).

### RRS-based methods describe a complex genetic structure of grape varieties at the clonal level

As acknowledged by the International Organization of Vine and Wine in resolutions OIV-VITI 424/2010 and OIV-VITI 564B-2019, a crucial conservation strategy to protect the world heritage of grapevine is based on the assessment of intra-varietal diversity and polyclonal selection (Resolution OIV-VITI 424/2010, 2010; Resolution OIV-VITI 564B-2019, 2019). In this study, the use of RRS-based analysis allowed the identification of a reliable set of clonal genetic variants to be employed in our genotyping experiment and the elimination of identical individuals within each variety studied. The presence of duplicated samples within fields, nurseries and germplasm repositories is quite common in clonally propagated species (Ipek et al., 2008; Ciftci et al., 2017; Orek et al., 2022; Selga et al., 2022). An exceptionally large number of identical genotypes was observed within the Aglianico Lasco population, where more than 70% of collected samples was duplicated. This redundancy is probably linked to the propagation history of clones, likely deriving from few homogenous plots of mother plants which were multiplied by local growers in a restricted cultivation area, under similar environmental pressures and selection criteria. After elimination of duplicated individuals, we were left with a set of 78 individuals that we employed in downstream investigations. We found that Camaiola, Greco B., Minutolo, Malvasia, and Nero di Troia split into separated populations. This finding indicates that these varieties are represented by clones with a genetically uniform profile. An exception was found in the population of Aglianico Lasco which separated into two groups called AL1 and AL2. To shed lights on the genotypic identity of each sub-population of Aglianico Lasco, we performed a comparative analysis based on microsatellites using, among others, the main red variety grown in Campania (Aglianico) with which Aglianico Lasco is generally matched and confused. The AL1 population included individuals genetically similar to all Aglianico biotypes (Taurasi, Taburno and Vulture), whereas AL2 grouped individuals with microsatellite profiles divergent from those of AL1. The high IBS values and the Kinship coefficient within AL2 (**Table S2**) suggest that these individuals have a more preserved genetic diversity, and therefore can be considered the true Aglianico Lasco, whereas AL1 individuals showed a greater variability, indicating that they may be homonyms (different cultivars named alike) of the most renowned cultivar Aglianico, whichhas at least three different biotypes inside.

Misattribution of names is frequently reported in grapevine (Crespan and Milani, 2001; De Lorenzis et al., 2014; Sunseri et al., 2018; Fanelli et al., 2021) and is considered the main cause of varietal confusion (Villano et al., 2022).

To explain the genetic diversity of the varieties under study, it is necessary to consider their origin and ancestry. Despite PCA plot did not reflect the geographical grouping, neighbour-joining tree clearly separated the Campanian from the Apulian varieties, indicating that the geographical origin could be one of the main drivers in defining varietal relationships in grapevine species, as observed in other studies (Fanelli et al., 2021).

Nero di Troia takes its name from the city of Troia in the Daunia region, where it is mainly cultivated. It derives from the spontaneous crossing between the French Bouteillan and the Bombino bianco, giving origin also to other Apulian varieties such as Bombino nero and Impigno (Miazzi et al., 2020; D’Onofrio et al., 2021). The reduced genetic variability between clones of Nero di Troia and their high genetic distance from the other Apulian varieties could further support the hypothesis that the province of Foggia represents a biodiversity hotspot for different crops (Conversa et al., 2018; Pisani et al., 2021); native varieties such as Nero di Troia could have been marginalised in those areas and therefore have underexplored gene pools that deserve to be studied to search for new and beneficial alleles. By contrast, the populations of Malvasia nera, Minutolo, AL1 and AL2 partially overlapped. This could be explained considering that these four varieties share a common ancestor, namely Visparola. Indeed, it has been reported that Malvasia Nera derives from a cross between Negroamaro and Malvasia bianca lunga, where Negroamaro is a descendent of Maiolica, an offspring of Visparola. In addition, the aromatic variety Minutolo derives directly from Visparola, also related to Aglianico (putative AL1 in our study) and its offspring; this confirms the centrality of Visparola in the origin of many grapevine varieties of Southern Italy (D’Onofrio et al., 2021). As the AL2 and AL1 populations partially overlap in the PCA plot, we hypothesise a parent-offspring relationship between Aglianico and Aglianico Lasco. Regarding Camaiola, PCA and F*_ST_* results showed that it differed more than anyone else from all the other varieties analysed. On the origin of this genetic distance, some hypotheses can be made based on the scarce information available. For example, the geographical distribution of Camaiola is restricted to the surroundings of Castelvenere city, where it is mostly cultivated, and has probably maintained a specific and uncontaminated genetic makeup. It should also be noted that DNA profiling provided evidence that Camaiola does not share close genetic links with any other Campania cultivar (Costantini et al., 2005; Villano et al., 2014). This suggests that Camaiola has been introduced relatively recently in Campania and supports the historical research by Selvaggio and Carlo (2015).

### A subset of divergent SNP loci is related to phenology and plasticity

In this work, the search for genetic differentiation at single loci yielded nearly 200 divergent SNPs and detected putative genes under selection. Camaiola displayed the greatest number of divergent loci, which supports its separation from all the other varieties in the F*_ST_* analysis. About 30% of the 200 divergent loci were in genes/QTLs involved in important phenological processes. For example, 12 divergent loci have been described as “switch genes” actively involved in the shift of berry developmental from immature to mature growth (Palumbo et al., 2014). This is a crucial physiological event that marks the ripening onset (called *veraison*) where numerous molecular, biochemical, and physiological changes occur that strongly impact the quality of wine (Conde et al., 2007; Fasoli et al., 2012; Castellarin et al., 2016; Fasoli et al., 2018). Among the divergent “switch genes” identified, VIT_203s0088g01250 and VIT_218s0001g14270 fell within phenology-related QTLs (Delfino et al., 2019) on chromosomes 3 and 18 responsible for phenotypes related to budding, flowering, soluble solid concentration, brix, and ripening. We also found signature of divergence in some genes involved in the regulation of multiple stress response and, therefore, putative players of plant adaptation to adverse biotic and abiotic conditions (Lee et al., 2010). For example, the arachidonic acid-induced DEA1 protein (VIT_202s0154g00280) in grapevine is involved in the phospholipid signalling processes and in the regulation of programmed cell death (PCD) in the scion/rootstock joining (Cookson and Ollat, 2013; Assunção et al., 2019). Notably, regulation of PCD is of paramount importance in plant-microbe interactions and is observed in many host resistance responses (Dickman and Fluhr, 2013). Greco B. and Nero di Troia were different at this locus. The drought tolerance reported for Nero di Troia (Antonacci, 2004) and its high susceptibility to powdery mildew compared to the low sensitivity to downy and powdery mildew of Greco B. (Rauscedo, 2011) are in agreement with these data.

We also found 14 divergent rootstock-responsive loci according to Corso et al. (2016). Among them, the protein CYCLIC NUCLEOTIDE-GATED CHANNEL 14 (CNGC14, VIT_204s0069g00790) and VIT_203s0038g02140. The former is known to be essential for the first step of auxin-induced Ca2+ signalling and growth inhibition in Arabidopsis root (Shih et al., 2015; Dindas et al., 2018); the latter is an auxin influx carrier protein, which is differentially expressed in graft interface tissues and possibly involved in directing the reconnection of vascular tissues (Yin et al., 2012; Cookson and Ollat, 2013). Finally, we identified an FKBP12-rapamycin complex-associated protein (VIT_203s0088g00450), with many divergent SNPs in Nero di Troia, which probably underwent selective pressure acting on this locus. The expression of VIT_203s0088g00450 was previously associated with bud dormancy (Shangguan et al., 2020), an essential adaptation process that allows temperate woody perennials to survive adverse environmental conditions during winter (Arora et al., 2003). Taken together, our data showed that the selection process shaped the genetic diversity of viticulturally attractive loci involved in fruit quality, growth and reproductive processes, the fine-tuning of which determines the fitness of fruit trees in changing climate conditions. We hypothesise that the molecular signatures we found could be the result of the selection operated by growers. It is possible that ripening time, sugar accumulation and stress response could have played a key role in determining whether a given cultivar was adapted to their specific local climate conditions. Interestingly, these functions appear strikingly similar to those in domesticated apple, peach, apricot and pear trees in which selective sweeps pointed to genes associated with growth cycle (Groppi et al., 2021) and fruit quality (Khan et al., 2014; Wu et al., 2018; Guan et al., 2021).

Our results provided evidence that a number of divergent loci overlapped with previously identified differentially methylated regions (DMR) in grapevine samples grown under changing environmental conditions (Xie et al., 2017). Among these we found the aforementioned VIT_203s0088g00450, and VIT_208s0007g07380, an HSP40 protein (also known as the DnaJ protein), which is known to contribute to cellular protein homeostasis under various biotic and abiotic stresses (Chen et al., 2021). Mounting evidence suggests that DNA methylation can affect local adaptation or plasticity by shaping the phenotypes that allow organisms to respond to their local environments (Richards et al., 2010). However, an active debate surrounds the question of whether (and to what extent) such epigenetic variations may either directly or indirectly affect local adaptation. Further experimental work is needed to corroborate these hypotheses.

## Conclusions

In this study, we used genome-wide SNP datasets generated by the GBS and ddRADSeq methods to assess the clonal diversity of six traditional grapevine varieties. We have shown that merging different SNP datasets is possible and valuable to study the inter- and intra-specific genetic diversity of grape populations. This provided that the same reference genome is used. Through such an approach, we have provided a repeatable framework to streamline future computational studies based on the retrieval of information from partial analyses performed at different times and with different techniques. Our results also demonstrated the value of advanced genomic methods in the study of population structure and synonymy/homonymy detection (as exemplified by Aglianico Lasco) as well in identifying possible recent introduced outgroup variety such as Camaiola for Campania. This is a crucial task in any germplasm management and conservation strategy. Finally, we identified several divergent SNP loci within genes involved in grapevine phenology and environmental adaptation. This evidence emphasises that some traits, such as those related to budding, flowering, and fruit quality could have played a key role in assessing whether a given cultivar was adapted to specific local climatic conditions.

## Data availability statement

The original contributions presented in the study are included in the article/**Supplementary Material**, further inquiries can be directed to the corresponding author/s.

## Author contributions

FT, CM, CV, and RA designed the experiment. CM, CV, PN, and RA established the grapevine collection. ES, SP, MM, FT, and ND’A carried out part of the bioinformatic and the genetic diversity analyses and detected loci under selection. VF and CV performed genetic diversity indices and SSR analysis. All authors were involved in the data interpretation. CV, FT, MM, and RA wrote the draft manuscript. CV, MM, and FT critically revised the manuscript for important intellectual content. DC, PN, CM, RA, ND’A, and FT finalised the latest version of the manuscript. All authors contributed to the article and approved the submitted version.

## Supplementary material

Supplementary Figure 1 SNP density plot showing the number of filtered SNPs in 1 Mb size windows for the 19 *Vitis vinifera* chromosomes in **(A)** Campanian and **(B)** Apulian datasets.

Supplementary Figure 2 Scatter plot showing the decay of linkage disequilibrium (r^2 = ^0.20) calculated for each of the six varieties.

Supplementary Figure 3 SNP density plot showing the number of SNPs in 1 Mb size windows for the 19 chromosomes of *Vitis vinifera*.

Supplementary Figure 4 Cross-validation error estimates for each value of K (i.e., number of sub-populations) tested.

Supplementary Figure 5
**(A)** Percentage cumulative variance for retained PCA eigen vectors. **(B)** Bayesian information criterion (BIC). **(C)** Scatterplot of the DAPC and DAPC F statistic.

Supplementary Figure 6 Distribution of divergent SNPs along the 19 chromosomes of *Vitis vinifera*.

Supplementary Table 1 List of clones under study. For each clone, the region of origin, the name of the variety, the clone identifier and the sampling site are reported. The clones selected after IBS analysis are in bold. The asterisks indicate the individual removed after filtering on merged dataset.

Supplementary Table 2 Identity-by-state (IBS) values and Kinship coefficients (Relatedness-PHI) calculated for the AL1 and AL2 populations.

Supplementary Table 3 List of divergent SNPs (*F_ST_* ≥ 0.80) falling into gene regions.

Supplementary Table 4 List of divergent SNPs derived from pairwise comparisons between clusters identified by ADMIXTURE and DAPC. Gene identifier, number of SNPs identified following pairwise comparison, gene function and functional group were reported.
