# Supplementary material for: Genetic diversity and signature of divergence in the genome of grapevine clones of Southern Italy varieties
Source: Front Plant Sci. 2023 Sep 13;14:1201287. doi: 10.3389/fpls.2023.1201287 (PMC10525710; doi:10.3389/fpls.2023.1201287)
Supplement: Supplementary Figure 1 — SNP density plot showing the number of filtered SNPs in 1 Mb size windows for the 19 Vitis vinifera chromosomes in (A) Campanian and (B) Apulian datasets. [file DataSheet_1.pdf]

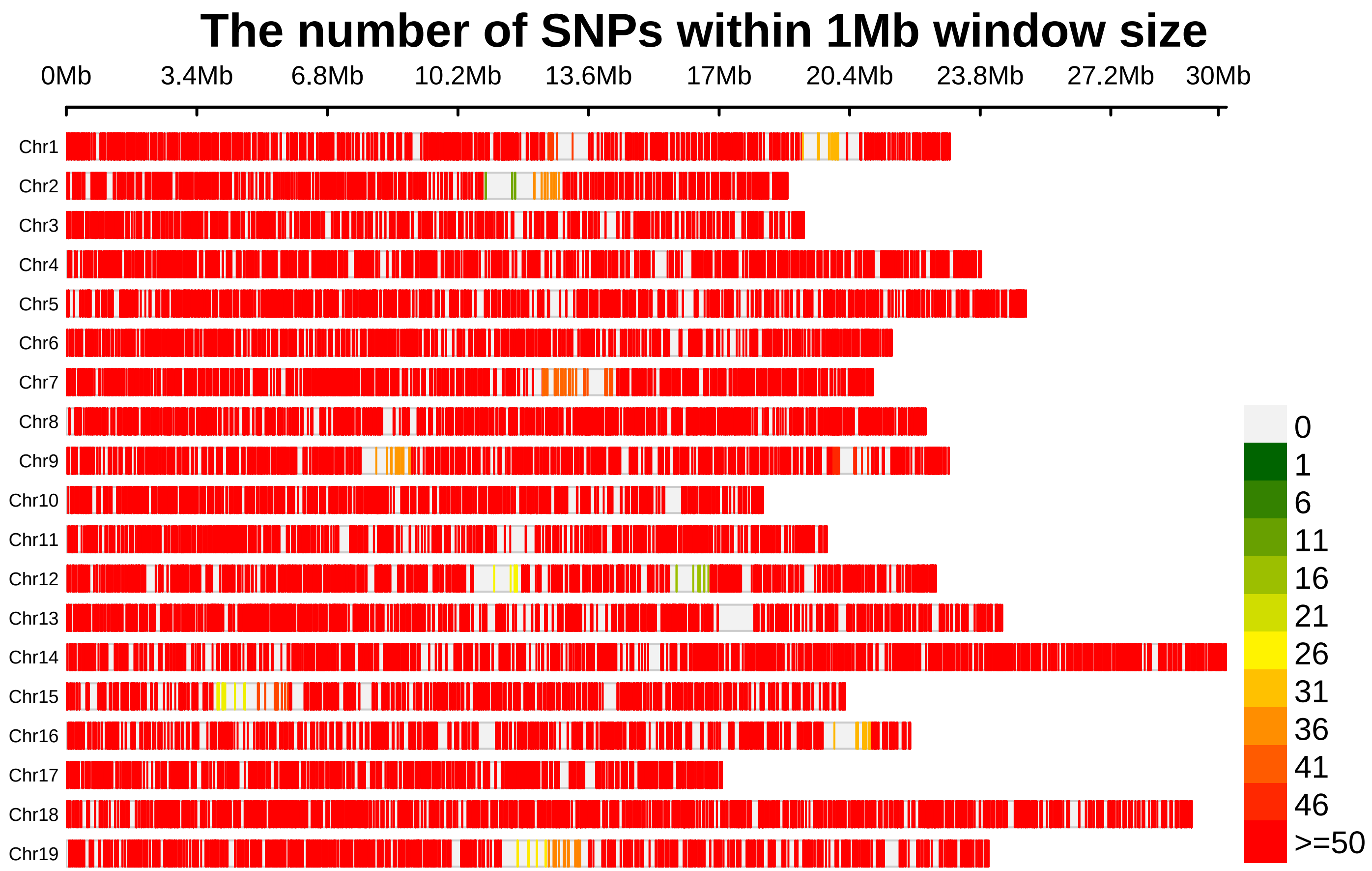
The number of SNPs within 1Mb window size
0Mb
3.4Mb
6.8Mb
10.2Mb
13.6Mb
17Mb
20.4Mb
23.8Mb
27.2Mb
30Mb
Chr1
Chr2
Chr3
Chr4
Chr5
Chr6
Chr7
Chr8
Chr9
Chr10
Chr11
Chr12
Chr13
Chr14
Chr15
Chr16
Chr17
Chr18
Chr19
0
1
6
11
16
21
26
31
36
41
46
>=50

Figure S1 A)

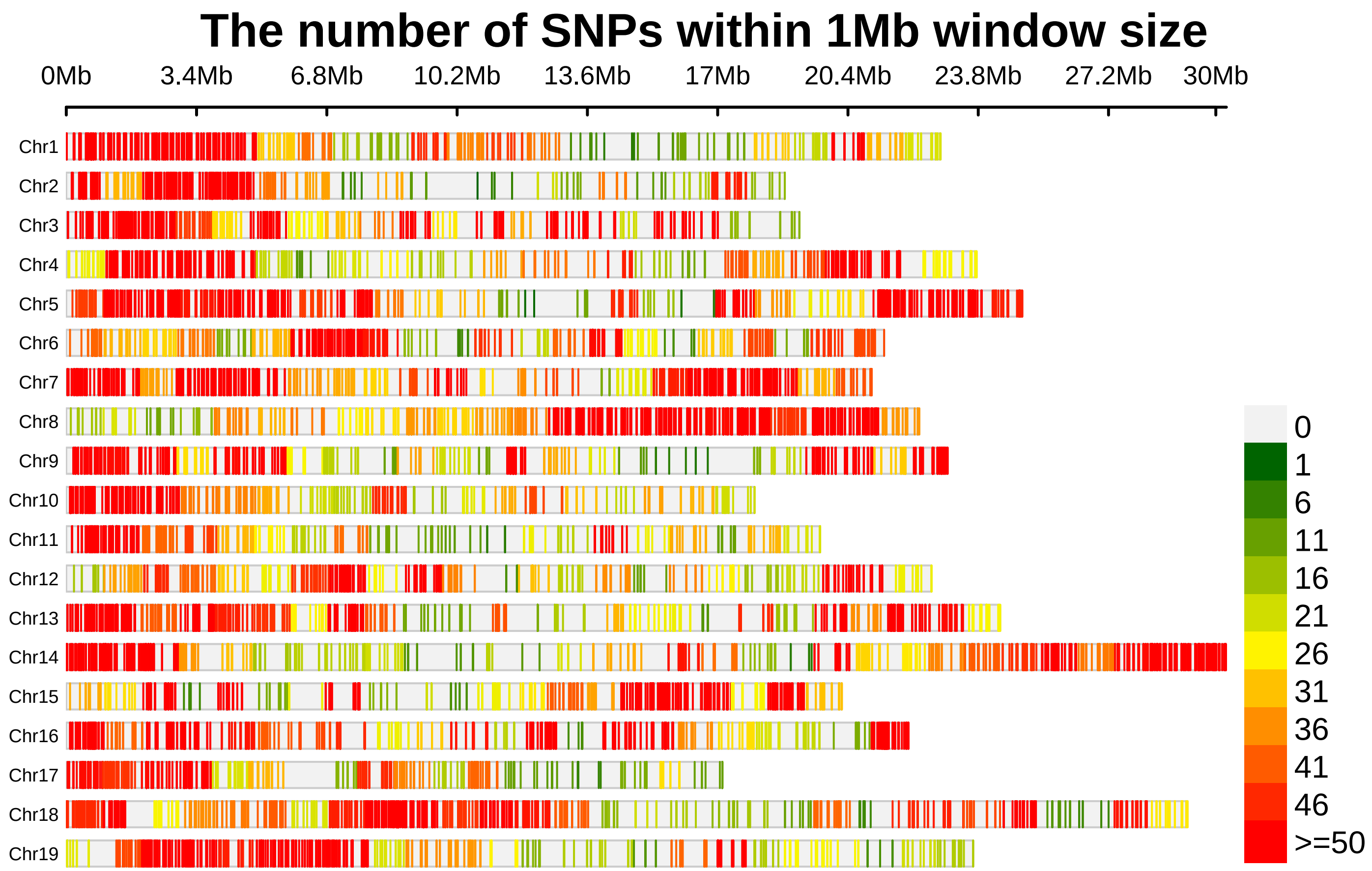
The number of SNPs within 1Mb window size
0Mb
3.4Mb
6.8Mb
10.2Mb
13.6Mb
17Mb
20.4Mb
23.8Mb
27.2Mb
30Mb
Chr1
Chr2
Chr3
Chr4
Chr5
Chr6
Chr7
Chr8
Chr9
Chr10
Chr11
Chr12
Chr13
Chr14
Chr15
Chr16
Chr17
Chr18
Chr19
0
1
6
11
16
21
26
31
36
41
46
>=50

Figure S1 B)
