# Supplementary figures and images for: Genetic diversity and signature of divergence in the genome of grapevine clones of Southern Italy varieties
Source: Front Plant Sci. 2023 Sep 13;14:1201287. doi: 10.3389/fpls.2023.1201287 (PMC10525710; doi:10.3389/fpls.2023.1201287)

Aglianico Lasco

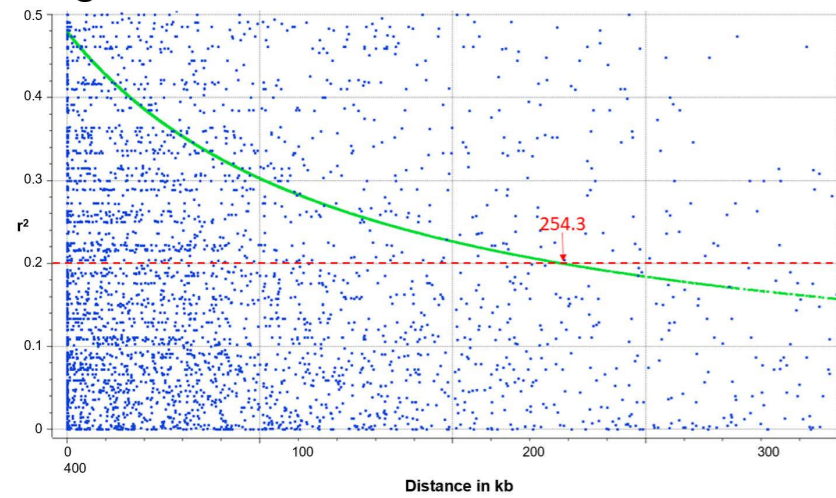

Minutolo

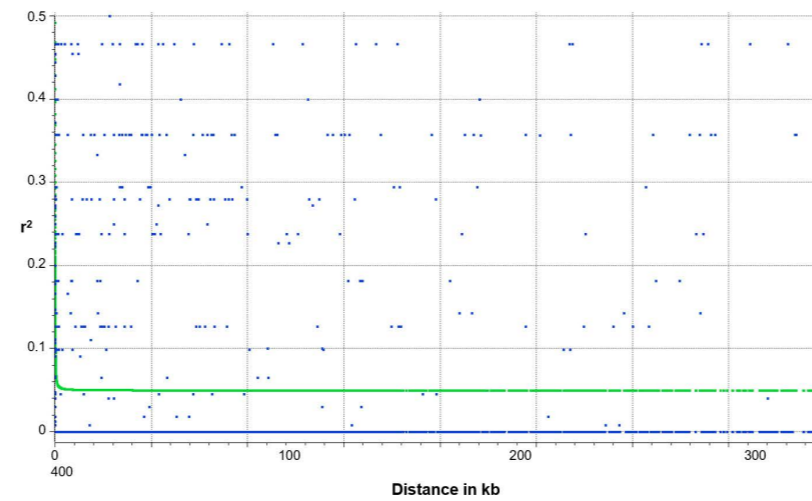

Greco B.

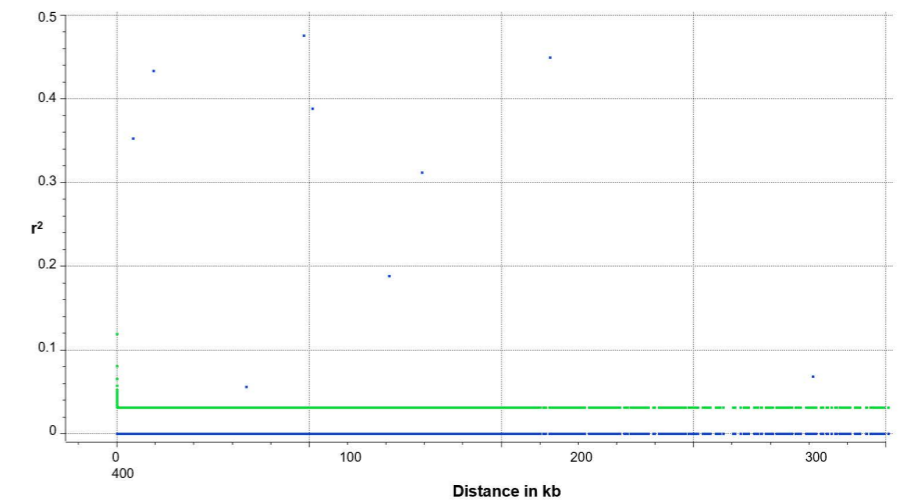

Malvasia nera

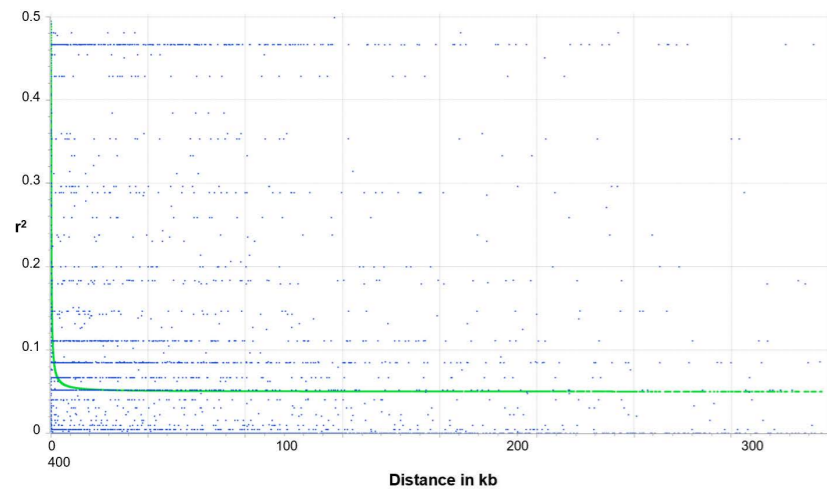

Nero di Troia

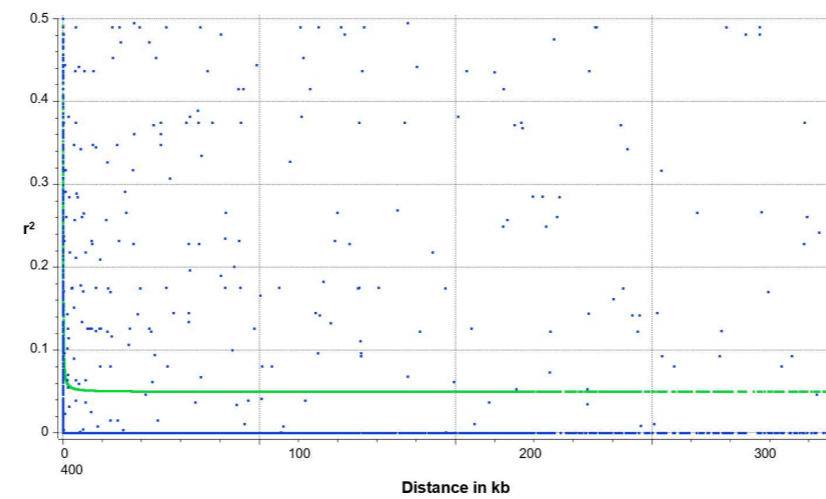

Camaiola

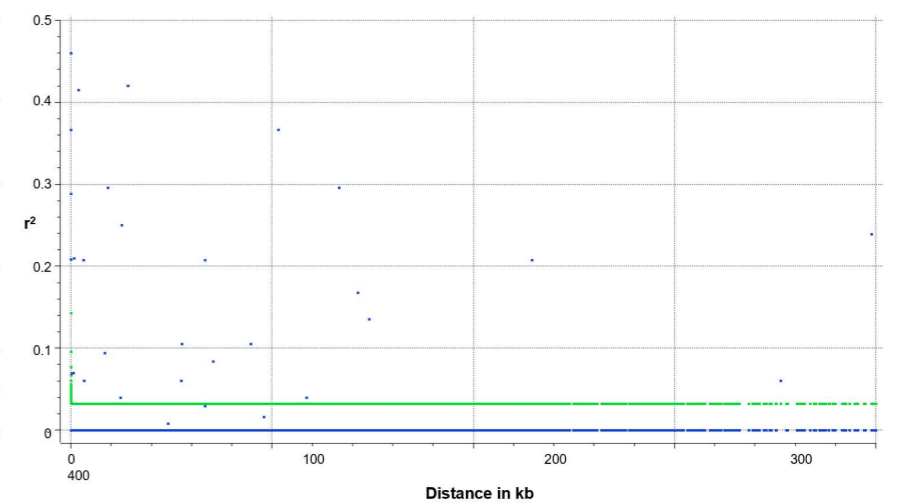

Figure S2

Supplement: Supplementary Figure 2 — Scatter plot showing the decay of linkage disequilibrium (r2 = 0.20) calculated for each of the six varieties. [file DataSheet_2.pdf]

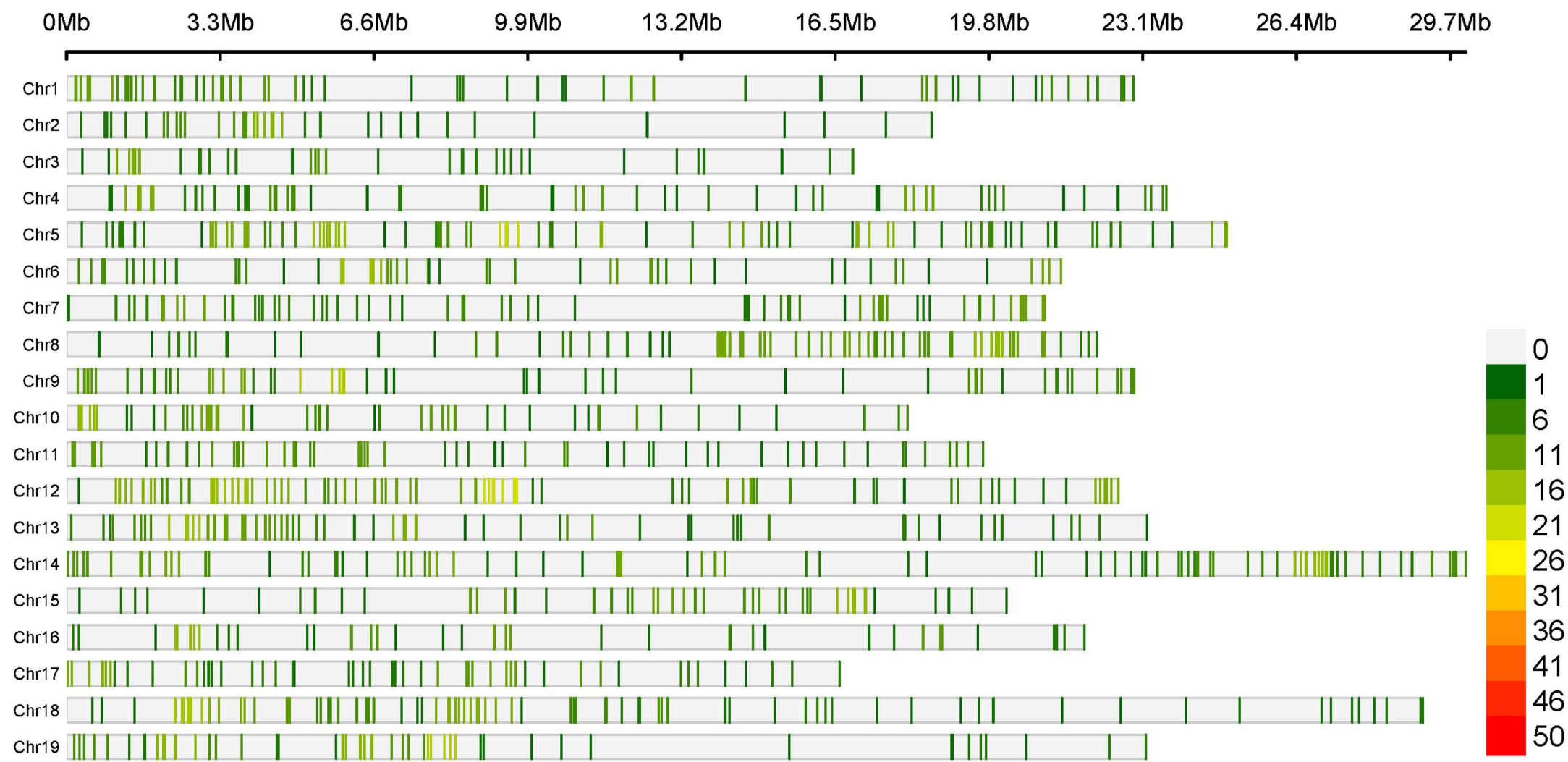

**Figure S3**

Supplement: Supplementary Figure 3 — SNP density plot showing the number of SNPs in 1 Mb size windows for the 19 chromosomes of Vitis vinifera. [file DataSheet_3.pdf]

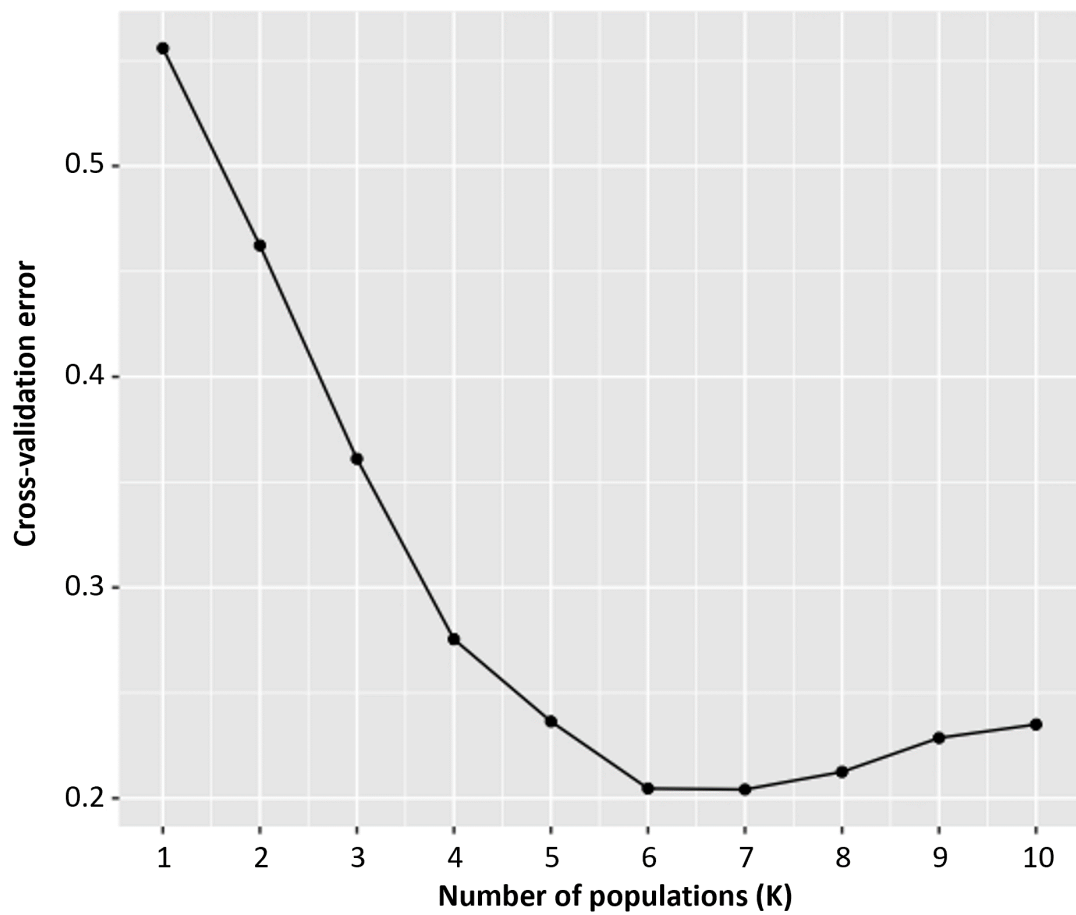

Figure S4

Supplement: Supplementary Figure 4 — Cross-validation error estimates for each value of K (i.e., number of sub-populations) tested. [file DataSheet_4.pdf]

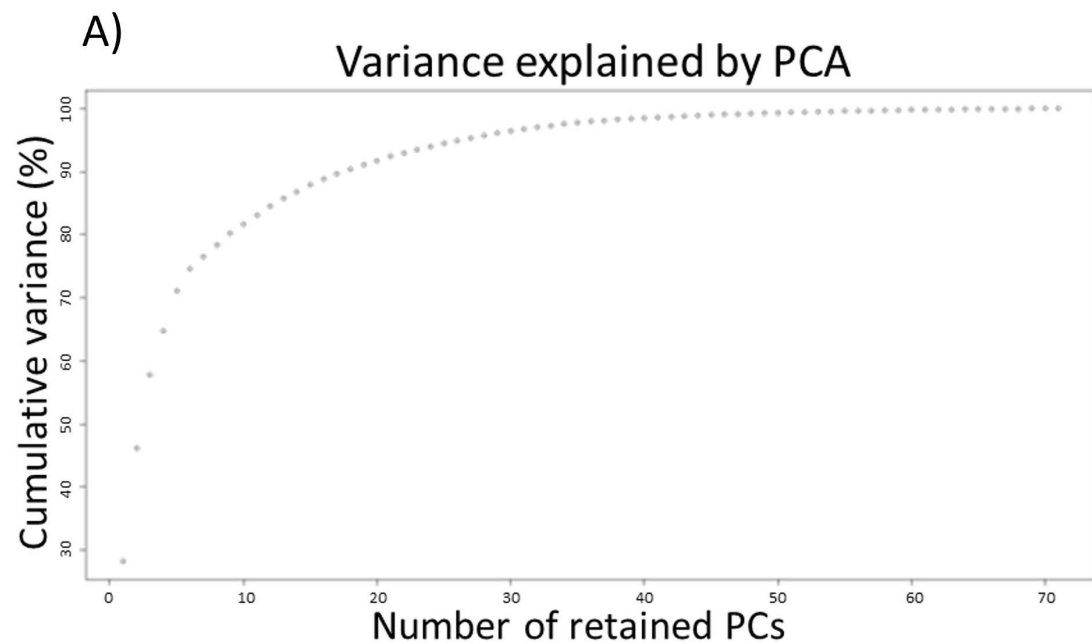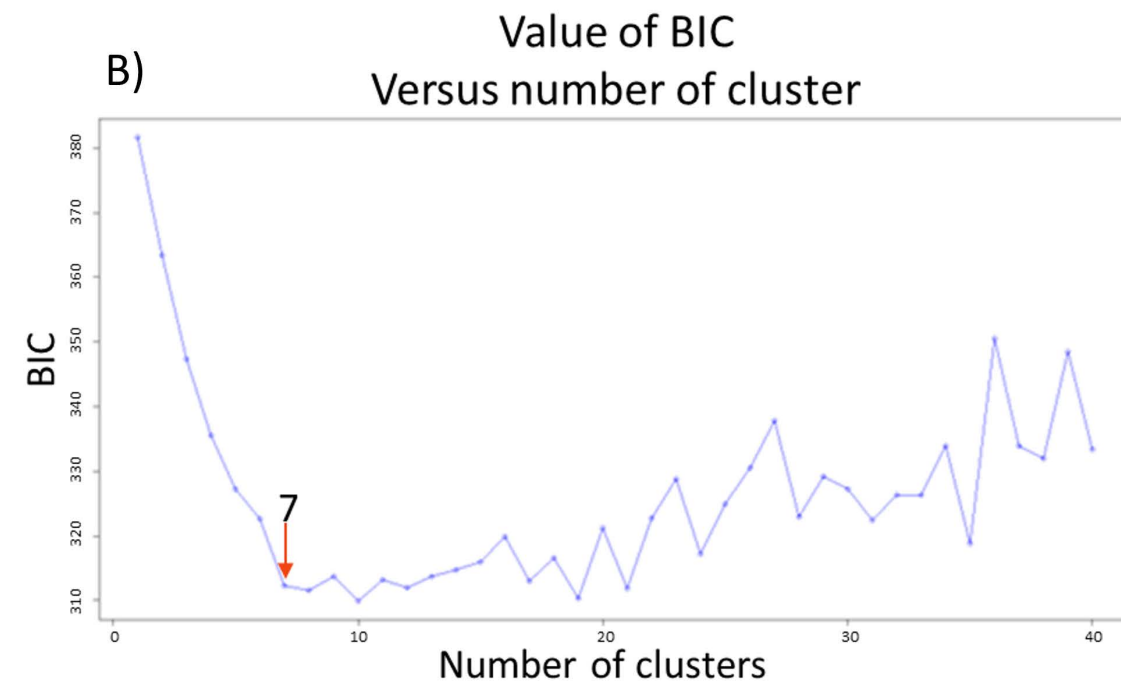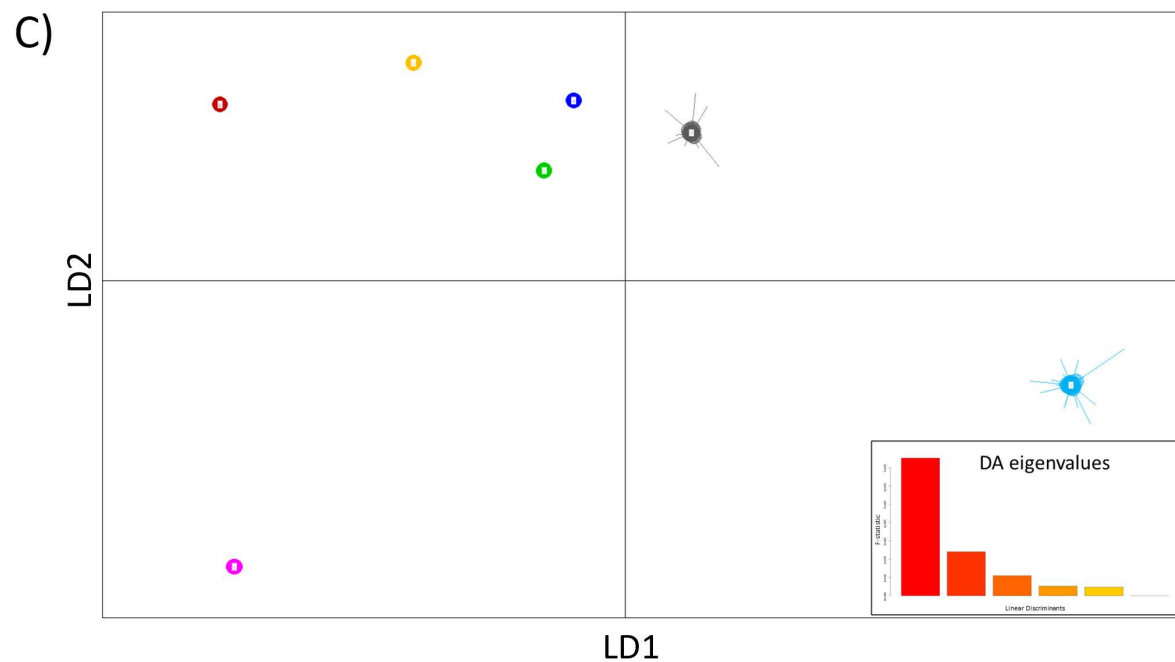

Figure S5

Supplement: Supplementary Figure 5 — (A) Percentage cumulative variance for retained PCA eigen vectors. (B) Bayesian information criterion (BIC). (C) Scatterplot of the DAPC and DAPC F statistic. [file DataSheet_5.pdf]

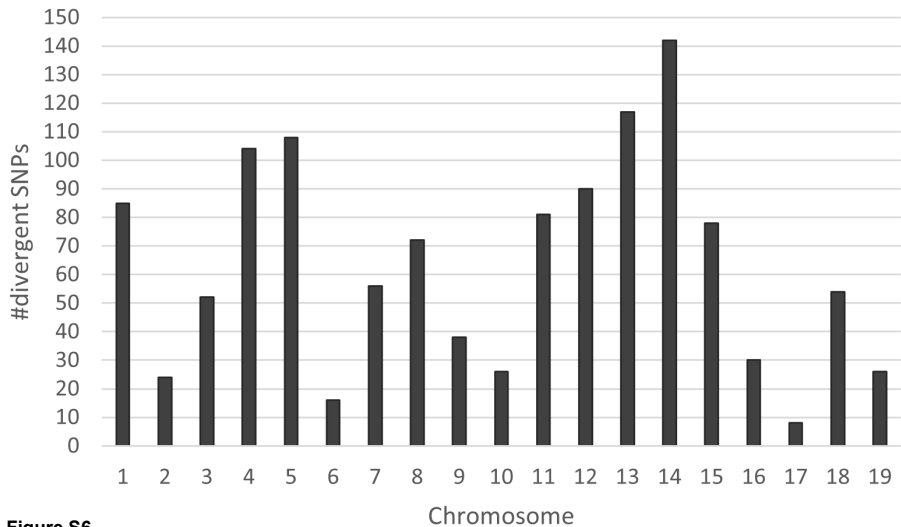

**Figure S6**

Supplement: Supplementary Figure 6 — Distribution of divergent SNPs along the 19 chromosomes of Vitis vinifera. [file DataSheet_6.pdf]
